# Grammatical ability and functional hearing in various listening conditions in 4–6-year-old children with prelingual unilateral hearing loss: a pilot study

**DOI:** 10.3389/fped.2025.1717513

**Published:** 2025-12-12

**Authors:** Ingrid Hedström, Ulrika Löfkvist

**Affiliations:** 1 Center for Clinical Research Sörmland, Eskilstuna, Sweden; 2 Department of Public Health and Caring Sciences, Uppsala University, Uppsala, Sweden

**Keywords:** unilateral hearing loss, grammatical ability, functional hearing, children, listening ability

## Abstract

**Objectives:**

This pilot study aimed to investigate receptive and expressive grammatical abilities and functional hearing in everyday listening conditions among 4–6-year-old children with prelingual unilateral hearing loss (UHL), compared with peers with normal hearing (NH). A further aim was to explore whether grammatical abilities were associated with functional hearing and background factors such as severity and side of hearing loss, or parental education.

**Methods:**

Eight children with UHL were recruited from hearing care clinics, and sixteen children with NH were recruited via social media. Data included questionnaires on hearing, development, and environment, and parent-reported functional hearing using PEACH+. Grammatical abilities were assessed with TROG-2 and the Swedish Gramba test. Group differences were analyzed with Mann–Whitney *U*-tests, and correlations were evaluated with Spearman's rank correlation.

**Results:**

Children with UHL scored lower than NH peers on language tests, though the differences were not statistically significant. They scored significantly lower on three of four parent-reported measures of functional hearing, particularly in noisy environments. Grammatical ability did not correlate with functional hearing. Across the cohort, higher parental education was associated with better grammatical outcomes. Within the UHL group, right-sided hearing loss was associated with poorer expressive grammar, and greater severity of hearing loss was associated with increased difficulty hearing in noise.

**Conclusions:**

Children with UHL demonstrated poorer functional hearing and tended to have slightly reduced grammatical abilities compared to peers with NH. Although the small sample size limits generalizability, these findings, together with previous research, suggest potential impacts of UHL on language development. The side and severity of hearing loss may influence outcomes, highlighting the need for further research and international consensus on assessment and management.

## Introduction

Receiving high-quality auditory input is crucial for auditory and spoken language development (1). Language and auditory perception are predominantly lateralized to the left hemisphere (2), with sound being primarily processed contralaterally to the receiving ear (3). Functional hearing includes sub-abilities like sound awareness, auditory feedback, sound source localization, auditory discrimination, short-term auditory memory, and linguistic auditory processing (4, 5). These abilities are essential for responding to various sounds and speech, especially in complex listening environments (6, 7). Children with more developed functional hearing abilities seem to use their language skills more effectively (8).

During the first year of life, neural networks are created as the child learns to distinguish and find patterns from hearing phonemes in the surrounding language (9). Grammatical knowledge emerges successively but increases markedly by two years of age (10). The development of grammar begins once the child has enough words to combine them into short sentences and/or begins using word conjugations (11, 12). To understand and express grammatical structures, like morphological conjugations, grammatical words, and syntactic relation; the child needs to decompose input into smaller linguistic units (13, 14). Prosodic difficulties, such as an inability to master syllable stress, may negatively affect expressive grammar (15). The frequency and familiarity of concepts also influence the acquisition of grammatical patterns (16). Sentence comprehension further depends on complex working memory, which is supported by language ability, attention control, and fluid intelligence (14). Fundamental grammatical ability is typically established by 4 years of age due to maturational changes in the brain (17). Grammatical competence, alongside lexical knowledge, is essential for broader language development, literacy, and academic achievement (18).

Beyond neurological and auditory aspects, environmental factors also influence language development. Biological and environmental risk factors may interact, contributing to language difficulties (19). As spoken language develops through social interaction, functional hearing is necessary for sufficient auditory input (1, 13). Rowe & Snow (20) emphasize that communication must be “responsive and engaging as well as linguistically adapted to the child's level and conceptually challenging” (p. 17). A study often cited is Hart & Risely (21), concluding that socioeconomically vulnerable children are generally less exposed to words, which have been questioned due to methodological issues (22). Bergelson et al. (23) did not find a correlation between the amount of talk from the parents and their socioeconomic status. They suggest that parental educational level might influence the use of more low-frequency words and complex sentence structures. Book reading is another important factor that impacts on the parents' language-rich interaction with their children (24), while higher exposure to screen media might negatively affect children's language development and functional hearing (8, 25). However, further research is needed to investigate the potential effects of the screen media content and contexts of usage (25).

Most Swedish children between ages 1 and 5 spend much of their time in preschool, which is characterized by high noise levels from children's speech and their activities (26). Noise is higher for children than teachers with mean levels reaching up to 82.6 dB (27). Preschool teachers' interactions with children play an important role in developing of academic talk; for example, the use of low-frequency words, domain-specific vocabulary, and more complex sentence structures (28, 29).

Children born with hearing loss risk auditory deprivation due to insufficient auditory input. Because of brain plasticity, early intervention can positively influence neurodevelopmental outcomes (30). The prevalence of permanent hearing loss in childhood is 3–4 per 1,000 children, and 20%–30% of these children have a unilateral hearing loss (UHL), depending on definition and detection rate (31–34). Children with UHL form a heterogeneous group with wide variability in etiology, severity of hearing loss, age at fitting of hearing aids (HA), usage of HA, and habilitation support (35, 36). Recent findings confirm this heterogeneity, showing etiologies ranging from congenital cytomegalovirus infection to cochlear nerve hypoplasia, each with different prognostic implications (37). Sound processing in UHL also tends to be more bilaterally distributed between left and right brain hemispheres (38, 39). There is still limited research on whether right- vs. left-sided UHL influences linguistic and auditory outcomes.

There is also a lack of high-quality studies about the consequences of prelingual UHL overall. Available results suggest auditory and linguistic vulnerabilities, although not all children show delays (35, 40, 41).

According to parental reports, children with hearing loss exhibit poorer functional hearing than peers with normal hearing (NH), especially in noisy environments, despite receiving early amplification (7). This is particularly relevant given the noisy preschool environment. Children with UHL also demonstrate poorer sound localization and speech perception than peers with NH (6, 42), and visual cues do not necessarily improve outcomes (43). Kutlu et al. (8) found that among children with cochlear implants semantics, morphology, syntax was significantly correlated with functional hearing, with consistent implant use and preschool attendance supporting development.

Children with UHL tend to exhibit poorer receptive and expressive language outcomes compared to population norms (44). Prosodic deficits may further impact expressive grammar (45). Early cochlear implantation in cases of prelingual profound UHL has been associated with typical linguistic and cognitive development (46, 47), whereas non-implanted children often show more heterogeneous outcomes (47). However, unilateral hearing alone may be insufficient to compensate for neural reorganization (48, 49), or to support verbal communication in complex listening environments (6).

There is an urgent need for more research to clarify the linguistic and auditory consequences of UHL in children, particularly in domains such as morphology and grammar, and to establish an evidence-based framework for pediatric hearing care (41, 50). A holistic approach is necessary, emphasizing everyday listening situations in terms of function, activity and participation (51). In summary, children with UHL may face risks to their language development and functional hearing. Therefore, there is an urgent need for further research to address existing knowledge gaps concerning grammar, functional hearing, and their interaction, both globally and within the Swedish context, which provides the rationale for the present study.

This pilot study aimed to investigate receptive and expressive grammatical abilities, as well as functional hearing in different listening conditions, in 4–6-year-old children with prelingual UHL, compared with age-matched peers with NH. Specifically, the study addressed the following research questions:

1. Do children with UHL differ from peers with NH in grammatical abilities, and if so, in which areas?
2. Do children with UHL differ from peers with NH in functional hearing, and if so, under which listening conditions?
3. Are grammatical abilities associated with functional hearing in children with UHL?
4. Are grammatical abilities or functional hearing related to background factors, such as side and severity of hearing loss or parental educational level?

## Materials and methods

The study was approved by Swedish Ethical Review Authority (Dnr. 2023-00849-01).

### Classification of hearing loss

The World Health Organization (WHO) defines UHL as a 4-frequency pure tone average (PTA) threshold of <20 dB in the better ear and ≥35 dB in the worse ear (52). In Swedish clinical practice, however, UHL may also be diagnosed when hearing thresholds fall within the range of mild hearing loss (20–<35 dB), moderate (35–<50 dB), moderately severe (50–<65 dB), severe (65–<80 dB), profound (80–<95 dB), or complete (≥95 dB). Type of hearing loss refers to whether it is sensorineural conductive, mixed, or unspecified (52), and whether it is right- or left-sided.

### Participants

Eight children with prelingual UHL (4.0–6.2 years; four females, four males) and 16 children with NH (4.2–6.6 years; eight females and eight males) participated. The UHL group was recruited through hearing care clinics in the Mideast Region of Sweden; the NH group was recruited from the local community through social media announcements. Written informed consent was obtained from caregivers prior to participation.

All children with UHL were identified through universal newborn hearing screening (UNHS). In the current study we had access to the participants most current audiogram (speech perception in quiet), and information related to hearing from the medical records.

Participant inclusion followed both the WHO classification for degree of hearing loss and the Swedish clinical definition of unilateral hearing loss. Accordingly, UHL was defined as a pure-tone average (PTA) ≥35 dB HL in the poorer ear and ≤20 dB HL in the better ear (53, 54), and was classified as moderate, moderately severe, or severe according to WHO criteria (52).

Age at diagnosis and HA fitting varied; HA usage was reported by parents (see Table 1). Three children (IDs 2, 3, 5) showed evidence of hearing loss in medical/audiogram records following UNHS but did not receive an official diagnosis until later for reasons that were not specified by parents. In most cases, hearing aid fitting occurred several months to years after the diagnostic hearing assessment. One child received a hearing aid shortly after diagnosis, whereas in another case the hearing aid was fitted approximately one year before the formal diagnostic assessment. One child with hearing loss >80 dB HL was fitted with a BAHA but uses it only intermittently, indicating that actual device use may influence functional benefit. Air-conducted thresholds were obtained for frequencies between 125 and 8,000 Hz (most commonly 250–8,000 Hz), and PTA were calculated as the mean of thresholds at 500, 1,000, and 2,000 Hz, representing the speech frequency range (Table 1).

**Table 1:** Demographic and hearing characteristics of children with unilateral hearing loss.

ID	Age (Y;M)	Sex	Mono-bilingual	Side of UHL	Type	NH ear (PTA)	UHL ear (PTA)	Detected	Age at Diagnosis (Y;M)	Age at HA fitting (Y;M)	HA	Usage of HA
1	4;0	Boy	M	Right	Sensorineural	20 dB^a^	56 dB	UNHS	0;2	0;4	BTE	Sporadic
2	4;0	Boy	M	Right	Sensorineural	20 dB^a^	61 dB	UNHS	3;11	2;11	BTE	Consistently
3	4;9	Girl	B	Left	Unspecified	20 dB^a^	44 dB	UNHS	2;6	3;10	BTE	Often
4	4;10	Boy	M	Right	Sensorineural	20 dB^a^	43 dB	UNHS	0;2	3;11	BTE	Consistently
5	5;1	Girl	M	Left	Sensorineural	15 dB^a^	63 dB	UNHS	3;5	4;2	BTE	Never
6	5;4	Girl	M	Right	Sensorineural	25 dB^a^	83 dB	UNHS	0;4	3;10	BTE	Sporadic
7	6;0	Girl	B	Right	Unspecified	24 dB	56 dB	UNHS	0;1	3;5	BTE	Consistently
8	6;2	Boy	M	Left	Sensorineural	8 dB	>80 dB	UNHS	0;5	1;3	BAHA	Sometimes

B, bilingual; M, monolingual; UNHS, universal newborn hearing screening; HA, hearing aid; BTE, behind the ear; dB, decibel; BAHA, bone-anchored hearing aid; PTA, pure tone average; Y;M, years; months.

a Screening data; actual hearing thresholds may hear better.

All NH children passed both UNHS and the four-year hearing screening at the Children's Healthcare Center. Inclusion criteria for both groups required at least one Swedish-speaking parent; two children with UHL were bilingual. All children attended preschools where Swedish was the only language used. Children with known additional disorders (e.g., developmental language disorder or other neurodevelopmental diagnoses) were excluded based on parent report.

Nonverbal cognitive ability was assessed using Raven's Progressive Matrices 2; no significant difference between groups (U = 57, *p* = .697). Based on parental reports, both groups had similar socio-economic backgrounds, including parental educational levels, and similar language stimulation environments (reading habits, singing activities, screen time usage) across groups (see Supplementary Appendix A).

### Materials

#### Background information

Parents completed a questionnaire addressing the child´s hearing, general development, and environmental factors. For children with UHL, questions included type and severity of hearing loss, age at detection, and age at HA fitting for children with NH, parents reported whether the child passed UNHS and the four-year screening at Childreńs Healthcare Center. Questions about general development included language and motor development, and occurrence of neurodevelopmental disorders. Environmental factors included:

a. Parent´s highest education level (1 = university degree, 2 = upper secondary school, 3 = elementary school),
b. Reading habits (1 = no regular reading, 2 = regular reading),
c. Average daily screen time (1 = 0–29 min, 2 = 30–59 min, 3 = 60–89 min, 4 = 90+ minutes), and
d. Heredity for neurodevelopmental disorders (1 = no, 2 = yes).

#### Raven's progressive matrices 2

This test assesses general non-verbal cognitive ability and was used to ensure a comparable cognitive level between groups. For children aged 4;0–8;11 years, it includes 36 items with a 30-min limit (55).

#### Swedish Gramba test

The Grammar test for children (Gramba) Test is a standardized assessment of Swedish expressive grammar for children aged 3–6 years (56). It takes approximately 20 min and evaluates:

○ Verb morphology (present/past tense, modal + infinitive, have/has + past participle)
○ Nominal morphology (plural, definite/indefinite articles, congruence, and genitives), and
○ Syntax (negation in/main/subordinate clauses, word order in topicalization).

Assessment uses sentence continuation and completion tasks. Maximum score is 44 points, which can be divided into verb, nominal and syntax sub-scores. Scores were converted into stanine values for comparison (see Table 2). Higher scores indicate better mastery of Swedish grammar. The test is standardized, age-appropriate, and has demonstrated construct validity for assessing expressive grammar and shows acceptable internal consistency across subscales, supporting its reliability as a measure of preschool children's grammatical abilities (56).

**Table 2:** Results for the language measures related to the test norms.

Language measures	Participants	
	Children with UHL *n* = 8	Children with NH *n* = 16
Gramba, stanine		
Median	2	4
95% confidence interval	[1, 7]	[3, 5]
Standard deviation	2, 5	1, 7
Min	1	2
Max	7	8
Gramba, *n* (%)		
Above average (Stanine 7–9)	1 (12, 5%)	3 (18, 75%)
Average (Stanine 4–6)	2 (25%)	7 (43, 75%)
Below average (Stanine 1–3)	5 (62, 5%)	6 (37, 5%)
TROG-2, percentile		
Median	26	51, 50
95% confidence interval	[1, 87]	[14, 75]
Standard deviation	32, 7	31, 8
Min	1	7
Max	87	99
TROG-2, *n* (%)		
+3 SD (Percentile 99)	0 (0%)	1 (6, 25%)
+2 SD (Percentile 91–98)	0 (0%)	1 (6, 25%)
+1 SD (Percentile 70–90)	2 (25%)	3 (18, 75%)
Average (Percentile 30–69)	2 (25%)	6 (37, 5%)
−1 SD (Percentile 16–29)	1 (12, 5%)	1 (6, 25%)
−2 SD (Percentile 2–15)	2 (25%)	4 (25%)
−3 SD (Percentile 0.1–1)	1 (12, 5)	0 (0%)

#### Test for reception of grammar (TROG-2)

TROG-2 is a standardized test for assessment of receptive grammar for children aged 4–16 years, validated in Swedish (57, 58). The test takes about 20 min and consists of 20 blocks, each targeting a specific grammatical construction: A) Two elements, B) Negative, C) Reversible in and on, D) Three elements, E) Reversible SVO (Subject-Verb-Object), F) Four elements, G) Relative clause in subject, H) Not only X but also Y, I) Reversible above and below, J) Comparative/absolute, K) Reversible passive, L) Zero anaphor, M) Pronoun gender/number, N) Pronoun binding, O) Neither nor, P) X but not Y, Q) Postmodified subject, R) Singular/plural inflection, S) Relative clause in object, and T) Centre-embedded.

In each block, children are asked to point to the picture that correctly matches a sentence read aloud, assessing their comprehension of the targeted grammatical construction. One point is given if all four items in a block are correct; maximum score is 20. Scores were converted into percentile values for analysis (see Table 2). The test is designed to minimize the influence of other language-related factors, like auditory discrimination, limited vocabulary, or working memory capacity. TROG-2 has demonstrated reliability and validity for assessing receptive grammar in children, and Swedish norms are available for children aged 4–12 years (*n* = 650).

#### Parents' evaluation of aural/oral performance of children (PEACH+)

PEACH + is a parent-report questionnaire assessing childreńs functional hearing in quiet and noisy everyday situations, validated in Swedish (59, 60). An updated back-translated version of the new PEACH+ was used (61), which also focuses on ease of listening in different environments. Parents rate the frequency of behaviors over the past week and how these have occurred in quiet and noisy situations: 0 = Never (0%), 1 = A little (25%), 2 = Sometimes (26%–50%), 3 = A lot (51%–57%) and 4 = Always (76%–100%). In the estimation of how easy or hard these situations are for the child, the parents provided a rating of the ease: 0 = Very hard, 1 = Hard, 2 = Neutral, 3 = Easy and 4 = Very easy. Each of four sections has a maximum score of 20; higher scores indicate better functional hearing and easier listening.

### Procedures

Before the data collection, picture-based instructions explaining the test procedure for children, and questionnaires assessing background information and functional hearing were sent home to parents. For children with UHL, when parents were unable to fully provide hearing-related information, the test leader (first author) supplemented missing details using medical records. It turned out that all the parents had trouble to completely fill out the hearing-related information. They were uncertain about the sequence of events and could not provide specific details regarding their child's hearing loss. The child's most recent audiogram was obtained from medical records.

Questionnaire data (PEACH+) assessing the child's functional hearing, along with the test protocols for TROG-2 and Gramba, were collected. All test protocols, questionnaires, and audiograms were pseudonymized and assigned a code key, which was stored separately from temporarily collected personal data.

Each child participated in a single 45–60-min assessment during which each child was assessed by an experienced speech-language pathologist (SLP; the first author). Prior to testing, children were informed that they could request a break at any time if needed. No child in either group requested a break; however, movement between tests was encouraged for a few children when signs of restlessness were observed before continuing the assessment. During testing, the SLP was seated opposite the child, facing them.

### Data analysis

Data were analyzed using SPSS (Version 29). Descriptive statistics were calculated for all variables. Normality was assessed using histograms and the Shapiro–Wilk test, which showed several tendencies for skewed distribution. Due to non-normal distributions and small sample sizes, non-parametric tests were used for all analyses. Group comparisons were conducted using the Mann–Whitney *U*-test. Effect sizes were estimated with *r* (0.10 = small effect, 0.30 = medium effect, 0.50 = large effect) (62). Correlations between grammatical abilities, functional hearing, and background variables were analyzed with Spearman's rank correlation. The significance level was set at *p* < .05.

## Results

Descriptive and comparative results of grammatical ability between groups (UHL vs. NH) are presented first, including corresponding Swedish norm scores for each test. Next, comparisons of functional hearing between the groups are reported. Data were missing for one NH participant (ID 17) regarding the estimated ease of hearing in quiet and noisy situations. Significant group differences are illustrated using boxplots. Lastly, correlation analyses are presented, examining the relationships between grammatical ability and functional hearing within each group, as well as correlations between these measures and background variables across the entire cohort (UHL and NH), including hearing-related variables for children with UHL.

### Grammatical abilities

No significant group differences were observed in either receptive or expressive grammatical ability (*p* > .05). However, four out of eight children with UHL scored below average in receptive grammar (Table 2). In both groups, below-average receptive grammar tended to coincide with below-average expressive grammar. A higher proportion of children with UHL also scored below average in expressive grammar, with five of eight children affected, compared to the NH group (Table 2). No specific grammatical construction was found to be particularly easier or more difficult for either group.

### Functional hearing

Children with UHL differed from NH peers on the PEACH+ measure, with consistently lower mean scores observed for the UHL group (see Supplementary Appendix B). Significant group differences were found for functional hearing in noisy situations: children with UHL (Md = 13) scored lower than NH children (Md = 17 raw), *U* = 17, *p* = .003, *r* = 0.59.

Significant differences were also observed for estimated ease of listening in both quiet and noisy environments. In quiet situations, children with UHL (Md = 14) scored lower than NH children (Md = 19), U = 16.5, *p* = .003, *r* = 0.59. In noisy situations children with UHL (Md = 8.5) scored lower than children with NH (Md = 18), U = 9.5, *p* < .001, *r* = 0.68).

**Figure 1:**
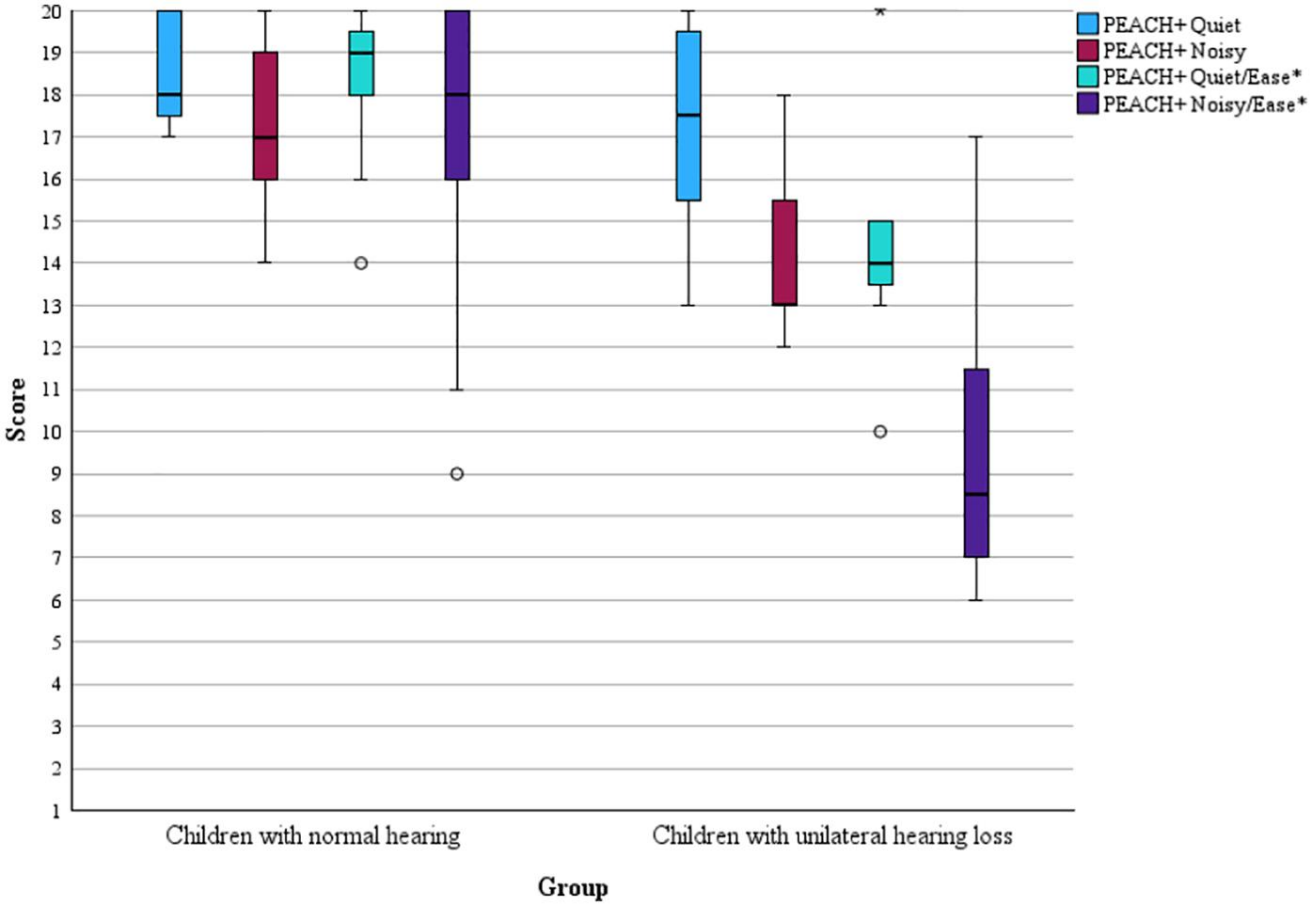
Group-level PEACH+ scores for UHL and NH. The boxplots display the median, outliers, lower and upper quartiles, and minimum and maximum values. *NH group: *n* = 15 due to missing data for one participant.

All significant comparisons demonstrated large effect sizes. No significant difference was found between groups for listening in quiet situations (*p* < .05). Scores for all four parameters are presented graphically in Figure 1.

### Correlation analyses of grammatical abilities, functional hearing and background variables

#### Grammatical ability and functional hearing

No significant correlations were found between receptive or expressive grammatical abilities and functional hearing in either group (*p's* > .05); see Supplementary Appendix C for the full correlation matrix.

#### Grammatical ability and parental education level

Across both groups, higher receptive and expressive grammatical scores were associated with higher parental education level (Table 3). A Maternal education level was also negatively correlated with regular reading irregularity, *r_s_* = −0.41, *n* = 24, *p* < 0.05, indicating more regular reading habits among children of more highly educated mothers.

**Table 3:** Correlation matrix between scores, background variables and hearing variables.

Variables	1. Gramba *Stanine*	2. TROG-2 *Percentile*	3. PEACH + *Quiet*	4. PEACH + *Noisy*	5. PEACH + *Quiet/Ease*	6. PEACH + *Noisy/Ease*	7. Education *Mother*	8. Education *Father*	9. Reading *Regularity*	10. Screen time *Average*	11. Hearing loss *PTA (dB) in UHL ear*	12. Hearing loss *Age at amplification*	13. Hearing loss *Side of UHL*
1. Gramba *Stanine*	1	.59*	.21	-.06	.07	.11	−.46*	−.46*	.18	.40	.08	.30	.90**
2. TROG-2 *Percentile*	.59*	1	−.01	−.00	−.02	.17	−.53*	−.50*	.22	−.12	−.01	.00	.50
3. PEACH + *Quiet*	.21	−.01	1	.69**	.74**	.47*	−.15	−.16	.07	.19	.05	−.17	.45
4. PEACH + *Noisy*	−.06	.00	.69**	1	.81**	.79**	−.05	−.10	−.21	−.16	.12	−.67	−.36
5. PEACH + *Quiet/Ease*	.07	−.02	.74**	.81**	1	.84**	−.08	−.27	−.09	.04	−.20	−.17	−.12
6. PEACH + *Noisy/Ease*	.11	.17	.47*	.79**	.84**	1	−.11	−.22	−.20	−.03	−.71*	−.01	−.68
7. Education *Mother*	−.46*	−.53*	−.15	−.05	−.08	−.11	1	.51*	−.41*	−.06	.32	.06	−.45
8. Education *Father*	−.46*	−.50*	−.16	−.10	−.27	−.22	.51*	1	−.31	−.03	.16	−.56	−.48
9. Reading *Regularity*	.18	.22	.07	−.21	−.09	−.20	−.41*	−.31	1	.23	–	–	–
10. Screen time *Average time*	.40	−.12	.19	−.16	.04	−.03	−.06	−.03	.23	1	.62	.16	.59
11. Hearing loss *PTA (dB) in UHL ear*	.08	−.01	.05	.12	−.20	−.71*	.32	.16	–	.62	1	−.13	.17
12. Hearing loss *Age at amplification*	.30	.00	−.17	−.67	−.17	−.01	.06	−.56	–	.16	−.13	1	.23
13. Hearing loss *Side of UHL*	.90**	.50	.45	−.36	−.12	−.68	−.45	−.48	–	.59	.17	.23	1

Data from both groups are merged, except for scores and background variables that are analyzed with the hearing variables linked to UHL group. Significate correlations that are relevant based on the purpose of the study are marked in color.

* *p* < .05.

** *p* < .001.

#### Grammatical ability and unilateral hearing loss

Receptive or expressive grammatical abilities were not significantly correlated with either the severity of hearing loss or age at hearing-aid fitting (*p's* > .05). However, expressive grammatical ability was strongly associated with the side of hearing loss, *r_s_* = 0.90, *n* = 8, *p* < .0.001, with children with right-sided UHL scoring lower on expressive grammar (Table 3).

#### Functional hearing and unilateral hearing loss

Functional hearing was not significantly correlated with age at hearing-aid fitting or side of hearing loss (*p's* > .05). Severity of hearing loss, however, correlated with greater reported difficulty hearing in noisy environments, *r_s_* = −0.71, *n* = 8, *p* < 0.05, (Table 3).

## Discussion

The aim of this study was to investigate the receptive and the expressive grammatical abilities, as well as the impact of functional hearing across different listening conditions, in 4–6-year-old children with prelingual UHL compared to peers with NH.

The results indicate that children with UHL experience significantly poorer functional hearing, especially in challenging listening environments, compared to children with NH. They also appeared to perform lower on measures of grammatical ability; however, these group differences did not reach statistical significance. In addition, the side of the hearing loss (right vs. left) may influence the manifestation of both linguistic and auditory difficulties. This finding warrants further investigation in larger, longitudinal studies.

### Differences in grammatical abilities

The findings revealed some differences in grammatical ability between children with UHL and those with NH. Although children with UHL generally scored lower on grammatical measures, these differences were not statistically significant, likely due to the small sample size. Nevertheless, a larger proportion of children with UHL had below-average scores compared to children with NH. Interestingly, four to six out of the 16 children with NH scored more than two standard deviations below the expected level based on normative data (see Table 2). While parents did not report any suspected developmental language disorders in these children, the test result suggest that some may have underlying language difficulties. This potential confound should be taken in account, as it may have influenced the group comparisons. However, confirming a language disorder requires a comprehensive assessment across multiple linguistic domains, which was beyond the scope of our study.

The overall tendency for children with UHL to obtain lower grammatical scores aligns with previous research, which has shown that this group is at risk of poorer receptive and expressive language outcomes, including grammatical skills (44, 47). Mastery of grammatical ability relies on the child's capacity to segment auditory input and to perceive relevant prosodic features (13–15). Unilateral hearing may not provide sufficient auditory input for such processes, particularly if neural changes associated with UHL limit access to high-quality auditory information (48, 49). Reduced input quality may, in turn, constrain opportunities for robust language development (1).

In the present study, a significant correlation was observed between right-sided UHL and poorer expressive grammatical ability, suggesting that the affected ear may influence linguistic development. Research specifically addressing the consequences of whether UHL is right- or left-sided remains limited. However, prior studies indicate that sound processing in individuals with UHL tends to be more bilaterally distributed across hemispheres compared to individuals with NH (38, 39).

One possible explanation for the current finding relates to the contralateral organization of auditory pathways: input from the right ear is predominantly processed in the left hemisphere, which is also central to language processing (2, 3). Consequently, right-sided UHL may reduce the quality or amount of auditory information reaching the language-dominant hemisphere, placing these children at risk for difficulties in expressive grammar. This interpretation, however, remains tentative and should be examines further in future research.

Although the group differences in grammatical ability did not reach statistical significance, clinically relevant differences were evident. Several children demonstrated poorer grammatical abilities than expected, which clinicians should take into account when planning management. This includes encouraging motivation for consistent HA use, and providing parents with guidance on strategies that support their child's language development.

All eight participating children with UHL were identified through UNHS, but variability was observed in the timing of diagnosis, the initiation of HA fitting, and the consistency of the HA use. Such variability reflects the lack of consensus in clinical management for this population and echoes broader discussions in the literature regarding best practices for supporting children with UHL (35, 36, 40, 41). Taken together, the finding that children with UHL demonstrate both poorer grammatical ability and reduced functional hearing underscores the need for more standardized, evidence-based approaches to intervention. Establishing clearer guidelines may help to ensure that the unique linguistic and auditory needs of children with UHL are systematically addressed.

The difficulties reported by parents in completing the questionnaire on hearing-related information raise concerns about their understanding of their child's hearing loss, the adequacy of the information they have received, and their awareness of its potential consequences. Early childhood is a critical period for establishing neural networks that support language processing, grammatical development, and the acquisition of core linguistic abilities (9–12, 17). Given the plasticity of the developing brain during this period, timely and consistent intervention may have a positive impact on neurodevelopmental outcomes (30). The heterogeneity in hearing-related variables observed in cohort, such as differences in age at diagnosis, etiology, HA fitting, and usage, may have influenced language outcomes, although the small sample size likely limited the ability to detect significant correlations. These findings underscore the importance of providing parents with comprehensive support to strengthen either understanding of UHL and to empower them in facilitating their child's auditory and linguistic development.

No significant correlations were found between environmental factors other than parental educational level. Parental education may influence children's language development through differences in word exposure, including both the quantity and complexity of vocabulary used at home (20–23). Other factors, like shared book reading and exposure to screen media, have also been suggested to shape children's access to language (24, 25), but no such associations were observed in our findings.

### Differences in functional hearing

Significant differences in functional hearing were observed between children with UHL and those with NH. Children with UHL scored significantly lower on three of the four parameters of functional hearing compared to peers with NH. This finding highlights the impact of UHL on functional hearing abilities, particularly in challenging listening conditions, such as noisy environments. This underscores the impact of UHL on everyday listening abilities, particularly in challenging environments like noisy settings. These difficulties may hinder children's ability to overhear speech, follow conversations, and actively participate in verbal interactions, which are crucial for social and language development. Importantly, these real-world challenges are not easily captured in standard audiological assessments conducted in controlled booth environments.

The lower scores in functional hearing scores in children with UHL, especially in noisy situations, are consistent with previous research (6, 7, 42). Although no significant correlations were found between functional hearing and most hearing-related variables, larger severity of hearing loss was associated with increased difficulty in noisy situations. These results suggest that difficulties in functional hearing, both in quiet and adverse listening conditions, are common among children with UHL, and that severity of loss further exacerbates challenges in everyday communication.

The development of auditory skills relies on receiving high-quality auditory input (1), which unilateral hearing may not sufficiently provide to compensate for potential neural changes in complex listening conditions (6, 48, 49). The participating children with UHL in our study attended preschool at levels comparable to their NH peers. Preschools are often characterized by high sound levels, with mean activity noise reaching approximately 82.6 dB for the children (26, 27). Children with UHL, who may be more vulnerable in auditory and linguistic domains (35), could therefore be at a disadvantage in such noisy environments (7). Moreover, visual cues may not reliably enhance speech perception in these settings (43), as they do often provide limited information about the content of the spoken communication.

### Grammatical Ability in Relation to Functional Hearing

No significant correlations were found between grammatical ability and functional hearing in either group, indicating that the non-significant differences in grammatical abilities between children with UHL and NH cannot be solely explained by variations in functional hearing. This suggests that additional factors may contribute to the linguistic and auditory development of children with UHL, warranting further investigation.

In a larger sample with well-defined subgroups based on hearing-related variables such as type and severity of hearing loss, and HA usage, correlations between grammatical and functional hearing abilities may emerge. The absence of significant correlations may also indicate that even children with relatively strong grammatical skills can experience difficulties in functional hearing. Over time, poorer functional hearing could impact grammatical development, particularly in school settings where exposure to low-frequency words and more complex sentence structures increases (28, 29).

The absence of significant correlations between grammatical ability and functional hearing in our study, in contrast to the findings of the Turkish study by Kutlu et al. (8), may be due to differences in sample sizes. Additionally, cross-linguistic differences between Swedish and Turkish grammar could have influenced the results. Children with UHL generally scored lower on both grammatical ability and functional hearing than controls in our study. Difficulties in functional hearing, especially in noisy environments, may persist even if grammatical structures are mastered, potentially limiting effective verbal communication (6). For instance, sentence comprehension relies on complex working memory and controlled attention (14), which may be affected by impaired functional hearing. Consequently, this can result in reduced exposure to academic language, including low-frequency words and complex sentence structures, often presented by preschool teachers and from highly educated parents (23, 28, 29). These nuances may not be fully captured by normative test designed for young children, highlighting the importance of considering real-life listening conditions when evaluating language development in children with UHL.

Even if correlations between grammatical abilities and functional hearing were not observed in this study, such relationships may emerge in larger samples or become more apparent as children reach school age. No participating children had diagnosed neurodevelopmental disorders. However, it is not possible to determine whether any participants may receive a diagnosis later. Similarly, children identified through UNHS may not present with neurodevelopmental disorders until older ages. Given that biological and environmental risk factors often co-occur and interact in the etiology of language difficulties (19), these considerations underscore the importance of early and preventive intervention (30).

### Heterogeneity in audiological characteristics and etiology

The heterogeneity in audiological characteristics within our UHL cohort, including side and severity of hearing loss, etiology, age at diagnosis, type of hearing device, and consistency of HA use, may contribute to variability in both functional hearing and language outcomes. Some children had congenital sensorineural loss, while others had unknown or acquired etiologies, which may differentially affect auditory input quality and subsequent language development. Although group differences in grammatical abilities did not reach statistical significance, individual children demonstrated clinically relevant deficits. These findings highlight that even within a relatively small and carefully selected cohort, audiological and etiological factors can influence language outcomes, underscoring the need for individualized assessment and management. Early identification, timely fitting of hearing aids, investigation of the cause of hearing loss, and support for consistent device use are crucial to optimize auditory exposure and facilitate language development. Recognizing this heterogeneity can help clinicians tailor interventions and guide parental counseling, ensuring that the unique needs of each child with UHL are addressed.

### Limitations

The selection of children with UHL reflects the reality of heterogeneity in hearing-related variables and the range of support provided by hearing care clinics (35, 36). Applying strict inclusion criteria further reduces the sample size, and conducting research with a larger cohort would require a multi-year study or recruitment across multiple hearing care regions in the country, which was not feasible in this study. A larger sample would also allow for greater variation in hearing-related factors, including type and severity of hearing loss, potentially revealing correlations between grammatical abilities, functional hearing, and hearing variables that were not detected here. Additionally, grammatical development is influenced by factors beyond auditory ability, like complex working memory (14), which was not investigated in the current study.

Additional heterogeneity could include children with non-Swedish-speaking parents or with co-occurring neurodevelopmental disorders, who were excluded from the present study. Despite this, the results indicate that children with UHL have poorer grammatical abilities and functional hearing even when “only” affected by UHL. In children with additional linguistic and socio-economic further vulnerabilities, the consequences of a UHL may be even more pronounced.

The selection of test materials was partly based on their common clinical usage in Sweden, ensuring that the results are relevant to everyday clinical practice. Standardized testing allows for the assessment of understanding and production of specific grammatical structures that may be difficult to evaluate through observation alone, and facilitates group comparisons using normative scores. However, this kind of assessments do not capture the functional impact of language difficulties for the individual. Currently, there is still no consensus regarding either the potential consequences of UHL or its optimal management in children (35, 40, 41). This may reflect the use of different language outcome measures across studies and the absence of tools that capture more subtle aspects of language or functional impact.

Hearing Aid (HA) usage was estimated based on parental report. Objective datalogging could provide a more accurate measure of actual usage time; however, this function is not always available and, when present, may be difficult to interpret depending on the period of data collection. Additional audiology appointments to obtain or clarify data logging were not feasible in this study due to organizational reasons and a shortage of audiologists. Children with profound UHL (>80 dB HL) may derive limited benefit from conventional hearing aids; the actual benefit also depends on the type of device and its consistent use, as illustrated by one participating child fitted with a BAHA who used it only intermittently.

The Speech Intelligibility Index (SII) provides a more precise estimate of functional hearing and potential benefit from amplification than the PTA. However, SII is not routinely measured during clinical follow-up for children with UHL in Sweden, and these data were therefore unavailable for the present cohort. Consequently, PTA was used as a proxy measure, which represents a limitation when interpreting potential aided speech perception.

### Future perspectives

The findings raise important questions about the long-term consequences of pediatric UHL and its implications for care. Parents of children with UHL had difficulties providing accurate information about their children's hearing loss, suggesting a need for qualitive research exploring parental knowledge and experiences with hearing healthcare. Such insights could inform the development and improvement of clinical practices.

Future multicenter, and inter-professional studies with larger sample sizes should longitudinally follow children with UHL in real-world settings. Inter-professional research should reflect the inter-professional management in hearing care, including audiologists, special educators, speech language pathologists, counselors and engineers. Research that captures everyday functioning, activity and participation (51) could provide outcome measures across hearing, language, communication, well-being, quality of life and school achievement, generating more comprehensive knowledge to guide management of children with UHL.

Brain imaging studies, using EEG or fMRI, could investigate neural organization in children with UHL, particularly comparing right- and left-sided cases and their potential effects on language and functional hearing outcomes. Further research is also warranted on potential compensatory or “camouflaging” behaviors in children with UHL. Addressing these questions may improve our understanding of the challenges associated with UHL and inform the development of more effective interventions and support strategies.

## Conclusions

Children with UHL tended to have slightly lower grammatical abilities compared to children with NH, although these differences did not reach statistical significance. In contrast, functional hearing abilities were significantly poorer in children with UHL, particularly in challenging listening conditions, such as noisy environments. Because of the modest sample size, the results by themselves cannot be generalized to the population. Taken together with previous studies, the results suggest that potential consequences for linguistic development in children with UHL cannot be ruled out. The side of the hearing loss (right vs. left) may also influence the appearance of linguistic and auditory difficulties, but this requires further investigation. National and international efforts are needed to develop professional consensus on the assessment and management of UHL in children.

## Data Availability

The datasets presented in this article are not readily available because the dataset presented in this article is not readily available due to participants lack of consent for its dissemination. Requests to access the datasets should be directed to ulrika.lofkvist@uu.se.

## References

[1] Flexer CA Madell JR Wolfe J Schafer EC. Why hearing is important in children. In: Madell JR Flexer C Wolfe J Schafer EC, editors. Pediatric Audiology: Diagnosis, Technology, and Management. 3rd ed. New York, NY: Thieme Medical Publishers (2019). p. 3–16.

[2] Price CJ. A review and synthesis of the first 20 years of PET and fMRI studies of heard speech, spoken language and reading. NeuroImage. (2012) 62(2):816–47. 10.1016/j.neuroimage.2012.04.062 22584224 PMC3398395

[3] Scheffler K. Auditory cortical responses in hearing subjects and unilateral deaf patients as detected by functional magnetic resonance imaging. Cereb Cortex. (1998) 8(2):156–63. 10.1093/cercor/8.2.156 9542894

[4] Stredler-Brown A Johnson CD. Functional Auditory Performance Indicators (FAPI). Phonak: Functional Auditory Performance Indicators (FAPI) (2001–2004).

[5] Ferreira K Moret ALM Bevilacqua MC Jacob R de S. Translation and adaptation of functional auditory performance indicators (FAPI). J Appl Oral Sci. (2011) 19:586–98. 10.1590/S1678-77572011000600008 22230992 PMC3973459

[6] Corbin NE Buss E Leibold LJ. Spatial hearing and functional auditory skills in children with unilateral hearing loss. J Speech Lang Hear Res. (2021) 64:4495–512. 10.1044/2021_JSLHR-20-00081 34609204 PMC9132156

[7] Persson A. Swedish Children with moderate hearing loss—on the importance of monitoring auditory and early speech development the first three years (Doctoral thesis, karolinska institute). (2020).

[8] Kutlu S Özkan HB Yücel E. A study on the association of functional hearing behaviours with semantics, morphology and syntax in cochlear-implanted preschool children. Int J Pediatr Otorhinolaryngol. (2021) 148:110814. 10.1016/j.ijporl.2021.110814 34214825

[9] Kuhl PK. Early language acquisition: cracking the speech code. Nat Rev Neurosci. (2004) 5(11):831–43. 10.1038/nrn1533 15496861

[10] Meylan SC Frank MC Roy BC Levy R. The emergence of an abstract grammatical category in children’s early speech. Psychol Sci. (2017) 28(2):181–92. 10.1177/0956797616677753 28074675

[11] Matthews D Tomasello M. Grammar. In: Reference Module in Neuroscience and Biobehavioral Psychology. Amsterdam: Elsevier (2017). p. 2–11. 10.1016/B978-0-12-809324-5.05819-3

[12] Casasola M Ransom A. Early semantic and grammar development. In: Benson JB, editor. Encyclopedia of Infant and Early Childhood Development. 2nd ed. Amsterdam: Elsevier (2020). p. 504–12.

[13] Clark EV. First Language Acquisition. 3rd ed. Cambridge, UK: Cambridge University Press (2016).

[14] Gillam RB Montgomery JW Evans JL Gillam SM. Cognitive predictors of sentence comprehension in children with and without developmental language disorder: implications for assessment and treatment. Int J Speech Lang Pathol. (2019) 21(3):240–51. 10.1080/17549507.2018.1559883 30712388 PMC6584051

[15] Sundström S Lyxell B Samuelsson C. Prosodic aspects of repetition in Swedish-speaking children with developmental language disorder. Int J Speech Lang Pathol. (2018) 21(6):623–34. 10.1080/17549507.2018.1508500 30557520

[16] Leclercq A-L Majerus S Jacob L Maillart C Prigent G. The impact of lexical frequency on sentence comprehension in children with specific language impairment. Res Dev Disabil. (2014) 35(2):472–81. 10.1016/j.ridd.2013.11.027 24361956

[17] Klein CC Berger P Goucha T Friederici AD Grosse Wiesmann C. Children’s syntax is supported by the maturation of BA44 at 4 years, but of the posterior STS at 3 years of age. Cereb Cortex. (2023) 33(9):5426–35. 10.1093/cercor/bhac430 36408641 PMC10152089

[18] Halliday MAK. Towards a language-based theory of learning. Linguist Educ. (1993) 5(2):93–116. 10.1016/0898-5898(93)90026-7

[19] Bishop DVM Snowling MJ Thompson PA Greenhalgh T, CATALISE consortium. CATALISE: a multinational and multidisciplinary Delphi consensus study. Identifying language impairments in children. PLoS One. (2016) 11:e0158753. 10.1371/journal.pone.0158753 27392128 PMC4938414

[20] Rowe ML Snow CE. Analyzing input quality along three dimensions: interactive, linguistic, and conceptual. J Child Lang. (2020) 47(1):5–21. 10.1017/S0305000919000655 31668157

[21] Hart B Risley TR. The early catastrophe: the 30 million word gap by age 3. Am Educ. (2003) 27(1):4–9.

[22] Purpura DJ. Language clearly matters; methods matter too. Child Dev. (2019) 90(6):1839–46. 10.1111/cdev.13327 31625601

[23] Bergelson E Soderstrom M Schwarz I-C Rowland CF Ramírez-Esparza N Hamrick LS Everyday language input and production in 1,001 children from six countries. Proc Natl Acad Sci U S A. (2023) 120:e2300671120. 10.1073/pnas.2300671120 38085754 PMC10756310

[24] Dickinson DK Griffith JA Michnick Golinkoff R Hirsch-Pasek K. How Reading books fosters language development around the world. Child Dev Res. (2012):602807. 10.1155/2012/602807

[25] Sundqvist A Barr R Heimann M Birberg-Thornberg U Koch F-S. A longitudinal study of the relationship between children’s exposure to screen media and vocabulary development. Acta Paediatr. (2024) 113(3):517–22. 10.1111/apa.17047 38014571

[26] Jónsdóttir V Rantala LM Oskarsson GK Sala E. Effects of pedagogical ideology on the perceived loudness and noise levels in preschools. Noise Health. (2015) 17(78):282–93. 10.4103/1463-1741.165044 26356370 PMC4900493

[27] McAllister AM Granqvist S Sjölander P Sundberg J. Child voice and noise: a pilot study of noise in day cares and the effects on 10 children’s voice quality according to perceptual evaluation. J Voice. (2009) 23(5):587–93. 10.1016/j.jvoice.2007.10.017 18456454

[28] Mashburn AJ Pianta RC Hamre BK Downer JT Barbarin OA Bryant D Measures of classroom quality in prekindergarten and children’s development of academic, language, and social skills. Child Dev. (2008) 79(3):732–49. 10.1111/j.1467-8624.2008.01154.x 18489424

[29] van Kleeck A. Distinguishing between casual talk and academic talk beginning in preschool years: an important consideration for speech-language pathologists. AJSLP. (2014) 23:724–41. 10.1044/2014_AJSLP-14-0032 25361384

[30] Kral A Kronenberger WG Pisoni DB O'Donoghue GM. Neurocognitive factors in sensory restoration of early deafness: a connectome model. Lancet Neurol. (2016) 15(6):610–21. 10.1016/S1474-4422(16)00034-X 26976647 PMC6260790

[31] Morton CC Nance WE. Newborn hearing screening—a silent revolution. N Engl J Med. (2006) 354(20):2151–64. 10.1056/NEJMra050700 16707752

[32] Fitzpatrick EM Whittingham J Durieux-Smith A. Mild bilateral and unilateral hearing loss in childhood: a 20-year view of characteristics, and audiologic practices before and after newborn hearing screening. Ear Hear. (2014) 35(1):10–8. 10.1097/AUD.0b013e31829e1ed9 24300117

[33] Fitzpatrick EM Al-Essa RS Whittingham J Fitzpatrick J. Characteristics of children with unilateral hearing loss. Int J Audiol. (2017) 56(11):819–28. 10.1080/14992027.2017.1337938 28639843

[34] Fitzpatrick EM Nassrallah F Gaboury I Whittingham J Fitzpatrick J. Trajectory of hearing loss in children with unilateral hearing loss. Front Pediatr. (2023) 11:1149477. 10.3389/fped.2023.1149477 37114003 PMC10126436

[35] Huttunen K Erixon E Löfkvist U Mäki-Torkko E. The impact of permanent early-onset unilateral hearing impairment in children—a systematic review. Int J Pediatr Otorhinolaryngol. (2019) 120:173–83. 10.1016/j.ijporl.2019.02.029 30836274

[36] Patel R Hoare DJ Willis KR Tabraiz S Bateman PK Thornton SK. Characterization of the treatment provided for children with unilateral hearing loss. Front Pediatr. (2023) 11:1197713. 10.3389/fped.2023.1197713 37559951 PMC10407268

[37] Aldè M Zanetti D Ambrosetti U Monaco E Gasbarre AM Pignataro L Unilateral sensorineural hearing loss in children: etiology, audiological characteristics, and treatment. Children. (2024) 11:324. 10.3390/children11030324 38539359 PMC10969491

[38] Burton H Firszt JB Holden T Agato A Uchanski RM. Activation lateralization in human core, belt, and parabelt auditory fields with unilateral deafness compared to normal hearing. Brain Res. (2012) 1454:33–47. 10.1016/j.brainres.2012.02.066 22502976 PMC3403813

[39] Parry LV Maslin MRD Schaette R Moore DR Munro KJ. Increased auditory cortex neural response amplitude in adults with chronic unilateral conductive hearing impairment. Hear Res. (2019) 372:10–6. 10.1016/j.heares.2018.01.016 29477243

[40] Walker EA. Evidence-based practices and outcomes for children with mild and unilateral hearing loss. Lang Speech Hear Serv Sch. (2020) 51(1):1–4. 10.1044/2019_LSHSS-19-00073 31913802 PMC7251591

[41] Laugen NJ Erixon E Huttunen K Mäki-Torkko E Löfkvist U. Newborn hearing screening and intervention in children with unilateral hearing impairment: clinical practices in three Nordic countries. J Clin Med. (2021) 10(21):5152. 10.3390/jcm10215152 34768671 PMC8584845

[42] Liu Q Wang Y Yang T Fan Y Hou B Chen Y Poor speech recognition, sound localization and reorganization of brain activity in children with unilateral microtia-atresia. Brain Imaging Behav. (2022) 16(1):78–90. 10.1007/s11682-021-00478-9 34245431 PMC8825362

[43] Lewis D Al-Salim S McDermott T Dergan A McCreery RW. Impact of room acoustics and visual cues on speech perception and talker localization by children with mild bilateral or unilateral hearing loss. Front Pediatr. (2023) 11:1252452. 10.3389/fped.2023.1252452 38078311 PMC10703386

[44] Carew P Shepherd DA Smith L Soh QR Sung V. Language and health-related quality of life outcomes of children early-detected with unilateral and mild bilateral hearing loss. Front Pediatr. (2023) 11:1210282. 10.3389/fped.2023.1210282 37645035 PMC10461396

[45] Sundström S. Prosodic and phonological ability in children with developmental language disorder and children with hearing impairment: in the context of word and nonword repetition (Doctoral thesis, Linköping university). (2018).

[46] Sangen A Dierckx A Boudewyns A Dhooge I Offeciers E Wouters J Longitudinal linguistic outcomes of toddlers with congenital single-sided deafness—six with and twelve without cochlear implant and nineteen normal hearing peers. Clin Otolaryngol. (2019) 44(4):671–6. 10.1111/coa.13347 31006171

[47] Arras T Boudewyns A Dhooge I Officiers E Philips B Desloovere C Assessment of receptive and expressive language skills among young children with prelingual single-sided deafness managed with early cochlear implantation. JAMA Netw Open. (2021) 4:e2122591. 10.1001/jamanetworkopen.2021.22591 34432009 PMC8387851

[48] Davies B Rattanasone NX Davis A Demuth K. Is one ear good enough? Unilateral hearing loss and preschoolers’ comprehension of the English plural. J Speech Lang Hear Res. (2021) 64:272–8. 10.1044/2020_JSLHR-20-00089 33285083

[49] Pittman AL de Diego-Lázaro B. What can a child do with one normal-hearing ear? Speech perception and word learning in children with unilateral and bilateral hearing losses relative to peers with normal hearing. Ear Hear. (2021) 42(6):1228–37. 10.1097/AUD.0000000000001028 33734172

[50] van Wieringen A Boudewyns A Sangen A Wouters J Desloovere C. Unilateral congenital hearing loss in children: challenges and potentials. Hear Res. (2019) 372:29–41. 10.1016/j.heares.2018.01.010 29395617

[51] Keidser G Naylor G Brungart DS Caduff A Campos J Carlile S The quest for ecological validity in hearing science: what it is, why it matters, and how to advance it. Ear Hear. (2020) 41(1):5S–19. 10.1097/AUD.0000000000000944 33105255 PMC7676618

[52] World Health Organization. World Report on Hearing. Geneva: World Health Organization (2021). Available online at: https://www.who.int/publications/i/item/9789240020481 (Accessed November 28, 2025).

[53] Bess FH Tharpe AM Gibler AM. Auditory performance of children with unilateral sensorineural hearing loss. Ear Hear. (1986) 7(1):20–6. 10.1097/00003446-198602000-00005 3949097

[54] Kuppler K Lewis M Evans AK. A review of unilateral hearing loss and academic performance: is it time to reassess traditional dogmata? Int J Pediatr Otorhinolaryngol. (2013) 77(5):617–22. 10.1016/j.ijporl.2013.01.014 23474216

[55] Raven J Rust J Chan F. Raven’s 2 Progressive Matrices, Clinical Edition. Pearson Clinical (2018). Available online at: https://www.pearsonclinical.asia/store/en/p/P100015139.html?utm_source=chatgpt.com (Accessed November 28, 2025).

[56] Nettelbladt U Hansson K. Gramba Grammatiktest för Barn—Helt Set. Lund: Studentlitteratur AB (2010).

[57] Bishop D. Test for Reception of Grammar, Version 2 (TROG-2). London, UK: Pearson Assessment (2003).

[58] Bishop D. Test for Reception of Grammar, Version 2 (TROG-2). Swedish Version. London, UK: Pearson Assessment (2009).

[59] Ching TYC Hill M. The Parents’ evaluation of aural/oral performance of children (PEACH) scale: normative data. J Am Acad Audiol. (2007) 18:220–35. 10.3766/jaaa.18.3.4 17479615

[60] Brännström R Bagatto M Scollie SD. Validation of the Swedish version of the parents’ evaluation of aural/oral performance of children (PEACH) rating scale for normal hearing infants and children. Int J Audiol. (2014) 53(10):693–8. 10.3109/14992027.2014.926497

[61] Löfkvist U Mäki-Torkoo E Johansson M Erixon E. (in manuscript). Validation of PEACH+ in a Swedish context.

[62] Cohen J. A power primer. Psychol Bull. (1992) 112:155–9. 10.1037/0033-2909.112.1.155 19565683

