# Supplementary material for: Grammatical ability and functional hearing in various listening conditions in 4–6-year-old children with prelingual unilateral hearing loss: a pilot study
Source: Front Pediatr. 2025 Dec 12;13:1717513. doi: 10.3389/fped.2025.1717513 (PMC12741133; doi:10.3389/fped.2025.1717513)
Supplement: Supplementary file 1 [file Supplementaryfile1.docx]

**Appendix 1.**

Table 1. Demographic information and language stimulation environments (reading habits, singing activities, and screen time usage) on individual level.

| **ID** | **Age** | **Sex** | **Education (Mother)** | **Education (Father)** | **Preschool** | **Reading** | **Singing** | **Screen time Onset** | **Screen time Average** | **Screen time Usage** |
| --- | --- | --- | --- | --- | --- | --- | --- | --- | --- | --- |
| **UHL** |  |  |  |  |  |  |  |  |  |  |
| **1** | 4;0 | Boy | University | Upper secondary school | 40 h/week | 20 min/day | None | Not reported | 0-29 min/day | Often with family member, sometimes alone |
| **2** | 4;0 | Boy | University | Upper secondary school | 40 h/week | 30-60 min/day | Daily | 2 years old | 60-89 min/day | Often with family member, sometimes alone |
| **3** | 4;9 | Girl | University | University | 45 h/week | 30 min/day | Sometimes | 2 years old | 30-59 min/day | Often alone, sometimes with family member |
| **4** | 4;1 | Boy | University | Upper secondary school | Yes | Yes | Yes | 2 years old | 0-29 min/day | Often alone, sometimes with family member |
| **5** | 5;1 | Girl | University | University | 40 h/week | 20 min/day | Daily | 1 year old | 90+ min/day | Often alone, sometimes with family member |
| **6** | 5;4 | Girl | Elementary school | Upper secondary school | 40 h/week | Rarely | Daily | 1 year old | 30-59 min/day | Often with family member, sometimes alone |
| **7** | 6;0 | Girl | Elementary school | Not reported | Preschool class | Yes | Yes | 2 years old | 30-59 min/day | Always with family member |
| **8** | 6;2 | Boy | University | Upper secondary school | Preschool class | 10 min/day | None | 3 years old | 60-89 min/day | Often alone, sometimes with family member |
| **NH** |  |  |  |  |  |  |  |  |  |  |
| **9** | 4;2 | Boy | University | University | 15 h/week | 10-15 min/day | Weekly | 1,5-2 years old | 60-89 min/day | Often with family member, sometimes alone |
| **10** | 4;2 | Girl | Upper secondary school | Upper secondary school | 45-50 h/week | None | None | From birth | 30-59 min/day | Often with family member, sometimes alone |
| **11** | 4;3 | Boy | Upper secondary school | Elementary school | 40 h/week | Sometimes | Daily | 2 years old | 90+ min/day | Often alone, sometimes with family member |
| **12** | 4;3 | Girl | University | University | 30 h/week | 20 min/day | Weekly | 1 year old | 30-59 min/day | Often with family member, sometimes alone |
| **13** | 4;4 | Girl | University | University | 40 h/week | 15-30 min/day | Weekly | 2 years old | 30-59 min/day | Often with family member, sometimes alone |
| **14** | 4;7 | Boy | University | University | Not reported | Weekly | Daily | 1 year old | 60-89 min/day | Often with family member, sometimes alone |
| **15** | 4;8 | Boy | University | University | 40 h/week | 5-10 min/day | None | Not reported | 60-89 min/day | Often alone, sometimes with family member |
| **16** | 4;9 | Boy | University | University | 35 h/week | 15-30 min/day | Daily | 2 years old | 0-29 min/day | Often with family member, sometimes alone |
| **17** | 4;10 | Girl | Upper secondary school | Upper secondary school | 15 h/week | 20 min/day | Weekly | 1,5 years old | 60-89 min/day | Often alone, sometimes with family member |
| **18** | 5;0 | Boy | Upper secondary school | University | 40 h/week | 20 min/day | Daily | 2 years old | 60-89 min/day | Often with family member, sometimes alone |
| **19** | 5;4 | Boy | University | Not reported | 40 h/week | 20 min/day | None | 2 years old | 90+ min/day | Often alone, sometimes with family member |
| **20** | 5;6 | Boy | University | Not reported | 45 h/week | 30-60 min/day | Yes | 1-2 years old | 60-89 min/day | Often with family member, sometimes alone |
| **21** | 5;10 | Girl | University | University | 35 h/week | 5-10 min/day | Weekly | 1 years old | 60-89 min/day | Often with family member, sometimes alone |
| **22** | 5;11 | Girl | Upper secondary school | Upper secondary school | 45-50 h/week | None | None | 3 months old | 30-59 min/day | Often with family member, sometimes alone |
| **23** | 6;3 | Girl | University | University | Preschool class | 10-25 min/day | Sometimes | 1,5-2 years old | 60-89 min/day | Often with family member, sometimes alone |
| **24** | 6;6 | Girl | University | University | Preschool class | 10 min/day | Weekly | 1 year old | 30-59 min/day | Often alone, sometimes with family member |
