# Supplementary material for: Grammatical ability and functional hearing in various listening conditions in 4–6-year-old children with prelingual unilateral hearing loss: a pilot study
Source: Front Pediatr. 2025 Dec 12;13:1717513. doi: 10.3389/fped.2025.1717513 (PMC12741133; doi:10.3389/fped.2025.1717513)
Supplement: Supplementary file 2 [file Supplementaryfile2.docx]

**Appendix 2.**

**Table 1.** Results of the functional hearing measures in both groups (UHL and NH).

|  | Participants | |
| --- | --- | --- |
|  | **Children with UHL**  n = 8 | **Children with NH** n = 16 |
|  |  |  |
| PEACH+ Quiet |  |  |
| Mean | 17,25 | 18,63 |
| 95 % confidence interval | [15,17, 19,33] | [17,95, 19,30] |
| Standard deviation | 2,49 | 1,26 |
| Min | 13 | 17 |
| Max | 20 | 20 |
|  |  |  |
| PEACH+ Noisy |  |  |
| Mean | 14,13 | 17,44 |
| 95 % confidence interval | [12,31, 15,94] | [16,48, 18,39] |
| Standard deviation | 2,17 | 1,79 |
| Min | 12 | 14 |
| Max | 18 | 20 |
|  |  |  |
| PEACH+ Quiet Ease* |  |  |
| Mean | 14,38 | 18,33 |
| 95 % confidence interval | [12,06, 16,69] | [17,36, 19,31] |
| Standard deviation | 2,77 | 1,76 |
| Min | 10 | 14 |
| Max | 20 | 20 |
|  |  |  |
| PEACH+ Noisy Ease* |  |  |
| Mean | 9,63 | 17,13 |
| 95 % confidence interval | [6,60, 12,65] | [15,23, 19,04] |
| Standard deviation | 3,62 | 3,44 |
| Min | 6 | 9 |
| Max | 17 | 20 |

*Notes:* ********Missing data from one participant with NH on the PEACH+ Quiet Ease and*

*PEACH+ Noisy Ease.*
