# Supplementary material for: Grammatical ability and functional hearing in various listening conditions in 4–6-year-old children with prelingual unilateral hearing loss: a pilot study
Source: Front Pediatr. 2025 Dec 12;13:1717513. doi: 10.3389/fped.2025.1717513 (PMC12741133; doi:10.3389/fped.2025.1717513)
Supplement: Supplementary file 3 [file Supplementaryfile3.docx]

**Appendix 3.**

**Table 1.** Correlation matrix between language scores and functional hearing scores in the NH group and in relation to normative data. *p <.05, **p <.001.

|  | **1. Gramba** *Stanine* | **2. Trog-2** *Percentile* | **3. PEACH+**  *Quiet* | **4. PEACH+**  *Noisy* | **5. PEACH+**  *Quiet/Ease* | **6. PEACH+** *Noisy/Ease* |
| --- | --- | --- | --- | --- | --- | --- |
| **1. Gramba** *Stanine* | 1 | .49 | -.12 | -.24 | -.04 | .11 |
| **2. Trog-2** *Percentile* | .49 | 1 | -.19 | -.08 | -.03 | .23 |
| **3. PEACH+**  *Quiet* | -.12 | -.19 | 1 | .80** | .83** | .56* |
| **4. PEACH+**  *Noisy* | -.24 | -.08 | .80** | 1 | .75* | .71* |
| **5. PEACH+**  *Quiet/Ease* | -.04 | -.03 | .83** | .75* | 1 | .75* |
| **6. PEACH+**  *Noisy/Ease* | .11 | .23 | .56* | .71* | .75* | 1 |

**Table 2.** Correlation matrix between language scores and functional hearing scores in the UHL group and in relation to normative data. *p <.05, **p <.001.

|  | **1. Gramba** *Stanine* | **2. Trog-2** *Percentile* | **3. PEACH+**  *Quiet* | **4. PEACH+**  *Noisy* | **5. PEACH+**  *Quiet/Ease* | **6. PEACH+** *Noisy/Ease* |
| --- | --- | --- | --- | --- | --- | --- |
| **1. Gramba** *Stanine* | 1 | .63 | .45 | -.30 | -.10 | -.61 |
| **2. Trog-2** *Percentile* | .63 | 1 | .04 | -.30 | -.63 | -.58 |
| **3. PEACH+**  *Quiet* | .45 | .04 | 1 | .55 | .63 | -.16 |
| **4. PEACH+**  *Noisy* | -.30 | -.30 | .55 | 1 | .69 | .18 |
| **5. PEACH+**  *Quiet/Ease* | -.10 | -.63 | .63 | .69 | 1 | .43 |
| **6. PEACH+**  *Noisy/Ease* | -.61 | -.58 | -.16 | .18 | .43 | 1 |
